# Telehealth and 30-Day Readmissions Among Heart Failure Patients During the COVID-19 Pandemic

**DOI:** 10.1093/geroni/igab046.1801

**Published:** 2021-12-17

**Authors:** Hanzhang Xu, Bradi Granger, Eric Peterson, Matthew Dupre

**Affiliations:** 1 Duke University School of Medicine, Duke University, North Carolina, United States; 2 Duke university School of Nursing, Durham, North Carolina, United States; 3 University of Texas Southwestern Medical Center, Dallas, Texas, United States; 4 Duke University School of Medicine, Durham, North Carolina, United States

## Abstract

This study examined whether outpatient follow-up within 14 days of discharge via telehealth visits are as effective as in-person visits for reducing 30-day readmission in heart failure (HF) patients. Using electronic health records from a large health system, we included HF patients (n=1,722) who were hospitalized during the period of March 15-July 15, 2020. Overall, 28.1% of patients received an early outpatient follow-up visit. Patients who received telehealth visits (n=119) were more likely to be older and live in areas with higher median household incomes than those with in-person visits (n=365). Thirty-day readmission rates were 20.5% during the COVID-19 period. Multivariate models showed that patients who received a telehealth (OR=0.36, 95%CI [0.23-0.56]) or an in-person (OR=0.42, 95%CI [0.31-0.57]) visit were less likely to be readmitted within 30 days compared with patients without an early outpatient follow-up. Telehealth visits were just as effective as in-person visits at reducing 30-day readmissions.

